# Whole Genome Sequencing and Comparative Genomics of Six *Staphylococcus schleiferi* and *Staphylococcus coagulans* Isolates

**DOI:** 10.3390/genes15030284

**Published:** 2024-02-24

**Authors:** Mohamed A. Abouelkhair, Stephen A. Kania

**Affiliations:** Department of Biomedical and Diagnostic Sciences, University of Tennessee College of Veterinary Medicine, Knoxville, TN 37996, USA; mabouelk@utk.edu

**Keywords:** *Staphylococcus schleiferi*, *Staphylococcus coagulans*, microbial taxonomy, epidemiology, comparative genomics, virulence genes

## Abstract

*Staphylococcus schleiferi* and *Staphylococcus coagulans*, closely related bacterial species within the *Staphylococcus* genus, present a challenge in classification and diagnosis due to their close genetic proximity and overlapping phenotypic features. Moreover, our understanding of the virulence mechanisms in staphylococcal species, beyond the extensively studied *Staphylococcus aureus*, remains limited, underscoring the importance of using comparative data to enhance our insights into virulence within these bacterial species. This study employed a comprehensive approach, utilizing comparative genomics, to identify genomic distinctions between *S. schleiferi* and *S. coagulans*, aiming to address the challenges in the accurate classification and diagnosis of these organisms and identify unique features. Whole genome sequencing was performed on six clinical isolates, and their genomes were compared to identify variations in gene content and virulence factors. De novo assembly and annotation revealed two samples as *S. coagulans* and four samples as *S. schleiferi*. Analysis of the core genomes revealed conserved regions crucial for defining species identity, while accessory genomic elements contained unique genes, possibly impacting the pathogenicity of the species.

## 1. Introduction 

*Staphylococcus schleiferi* is a Gram-positive bacterium within the genus *Staphylococcus*. It has the ability to cause infections in both veterinary and human medicine. *S. coagulans* has been identified as a commensal and opportunistic pathogen affecting primarily dogs, where it is frequently detected on the skin [1] and external ear canal [2,3]. Furthermore, it has been linked to external ear otitis [1,2] and pyoderma [2,3]. Although rare, there have been documented cases of *S. coagulans* producing opportunistic infections in immunocompromised humans [3,4]. The genetic similarity and common phenotypic traits of these two bacterial species have provided considerable challenges to correct classification and diagnosis [5,6]. Their clinical importance justifies the need for a better understanding of their genetic and pathogenic properties as well as the development of new methods of diagnosis, prophylaxis and treatment.

The former subspecies of *S. schleiferi*, *S. schleiferi* subsp. *coagulans,* and *S. schleiferi* subsp. *schleiferi*, have been reclassified as different species—*S. schleiferi* and *S. coagulans*, respectively [7]. This reclassification was based on genome-to-genome distance, average nucleotide identities, and partial 16S rRNA genes comparing *S. schleiferi* subsp. *coagulans* GA211^T^ with *S. schleiferi* subsp. *schleiferi* ATCC 43808^T^. A more extensive study highlighted the importance of genetic-based diagnostics in light of the failure of current, commonly used laboratory methods, including matrix-assisted laser desorption ionization–time of flight (MALDI-TOF), to distinguish them [6]. Even the coagulase test, which has historically been used to distinguish the (former) subspecies, has been shown to be unreliable for this purpose because of false reactions caused by pseudo-coagulase activity. Moreover, 16S rRNA sequencing is often used to distinguish bacterial species; however, it has been shown that these sequences in public databases contain a large number of nucleotide errors and misclassifications [8,9], and even 16S rRNA genes, though thought to be highly conserved, can have variability within a single species [10]. To address this problem, we employ comparative genomics techniques that can be used to elucidate the complexities of gene content, pangenomes, and dynamic genetic components, identifying distinguishing traits that can serve as robust classifiers. The identification of these genetic markers provides the basis for the development of cost-effective methods to both distinguish species and also to overcome the difficulties in accurate classifications of clinical isolates. 

In addition, this study helps fill a gap in our knowledge of staphylococcal pathogenicity pathways. Whereas numerous studies have been conducted on *S. aureus* [11,12,13,14,15,16,17] and *S. pseudintermedius* [18,19,20,21], the virulence mechanisms of closely related species such as *S. schleiferi* and *S. coagulans* remain largely unexplored. This study adds to our understanding of the pathogenic potential and mechanisms of the *Staphylococcus* genus through genomic analysis. Methicillin-resistance in *S. coagulans* and *S. schleiferi*, as with other staphylococci, is associated with an increasing trend toward multi-drug resistance and poses a significant treatment challenge [22,23,24]. Thus, it is essential that alternative treatments and prophylaxis be developed. Strategies for this process require the identification of virulence proteins, resistance mechanisms, and knowledge of strain conservation.

This study explores genomic variability in *S. schleiferi* and *S. coagulans* isolates. It addresses challenges in classification and diagnosis, examines virulence mechanisms within two important members of the *Staphylococcus* genus, and lays the groundwork for improved microbial identification and infectious disease management. These areas of research not only contribute to scientific understanding but also hold promise for advancements in clinical microbiology and patient care.

## 2. Materials and Methods

### 2.1. Bacterial Strains, Media, and Growth Conditions 

Bacteria propagated in this study included three *S. schleiferi* isolates (*S. schleiferi* 192, *S. schleiferi* 205, and *S. schleiferi* 214), two *S. coagulans* (*S. coagulans* 191 and *S. coagulans* 196) submitted to diagnostic laboratories from human cases in the USA, and the *S. schleiferi* type strain ATCC43808 isolated from a human patient in France and obtained from a culture collection. The bacteria were streaked on blood agar plates, and a single colony from each isolate was inoculated into 5 mL of sterile trypticase soy broth (TSB) (BD Biosciences, San Jose, CA, USA; Cat. no. RS1-011-21) and grown overnight with shaking at 225 rpm in a 37 °C incubator. 

### 2.2. DNA Extraction, Library Preparation, and Whole Genome Sequencing

DNA was extracted using the MasterPure DNA purification kit (Epicentre, Madison, WI, USA, cat. no. MCD85201) following the manufacturer’s protocol. The Nextera DNA sample prep kit (Illumina, Inc., San Diego, CA, USA) was used to prepare libraries for sequencing. Genomes were sequenced using the MiSeq platform (Illumina, Inc.) with two runs (2 × 75 bps) at the University of Tennessee Genomics Core facility. Sequences were trimmed using BBDuk and de novo assembled using Geneious Prime^®^ 2023 [25]. The quality of the assembled genomes was determined using the quality assessment tool for genomic assemblies (QUAST). [26]. The sequences were annotated with the NCBI Prokaryotic Genome Annotation Pipeline version 4.6 (https://www.ncbi.nlm.nih.gov/genome/annotation_prok, accessed on 17 October 2023) using the best-placed reference protein set with GenMarkS+.

### 2.3. Comparative Genomics Analysis

For whole-genome-based taxonomic analysis, the genome sequence data were uploaded to the Type (Strain) Genome Server (TYGS). This bioinformatics platform is available at https://tygs.dsmz.de, accessed on 17 October 2023 [27]. The analysis included methodological features for genome-based classification and nomenclature of prokaryotes described by Meier-Kolthoff et al. [28]. Information on synonymy, nomenclature, and associated taxonomic data was obtained using the LPSN (the List of Prokaryotic names with Standing in Nomenclature) which is available at https://lpsn.dsmz.de, accessed on 17 October 2023 [28]. The results were obtained from TYGS on 18 December 2023. The closest type strain genomes were determined using a sequential two-step process. First, using the MASH algorithm, a comparison was made with our genomes and all type strain genomes contained in the TYGS database [29]. Second, 10 closely related type strains were identified based on their 16S rRNA gene sequences. RNAmmer was used to extract them from the uploaded genomes [30], and each sequence was then subjected to BLAST analysis [31] against the 16S rRNA gene sequence of each of the type strains contained within the TYGS database. This approach, based on their bitscore, was used to find the 50 most similar type strains for each query genome. Subsequently, the Genome BLAST Distance Phylogeny (GBDP) approach with the algorithm coverage and distance formula d5 was used to determine precise distances [32]. The 10 type strain genomes closest to each of our genomes was thus determined. To detect phylogenomic inference, pairwise comparisons of genomes were made using GBDP, and intergenomic distances were inferred using the trimming algorithm and distance formula d5 [32]. For each analysis, a total of 100 replicates were determined, and the protein sequences of entire proteomes were used to conduct an additional GBDP phylogenomic analysis, allowing a robust resolution of the phylogeny of remotely related strains. Digital confidence intervals and DNA: DNA hybridization (DDH) values were determined using GGDC 4.0 with the recommended settings [28,32]. The intergenomic distances obtained from this analysis were used to produce a balanced minimum evolution tree with branch support inferred from 100 pseudo-bootstrap replicates using FASTME 2.1.6.1 with SPR postprocessing [10]. The trees were rooted at the midpoint [33] and visualized with PhyD3 [34]. The species clustering was performed using a 70% digital DNA: DNA hybridization (dDDH) radius around the 20 type strains as described previously [27]. Subspecies clustering was determined using a 79% dDDH threshold as described by Meier-Kolthoff et al. [35]. 

The genomes assembled from our isolates were compared against a set of complete genomes of *S. schleiferi* and *S. coagulans* downloaded from the NCBI database (Table 1) using Anvi’o 8 [36]. 

Average nucleotide identity (ANI) values were calculated using Anvi’o 8 [36] via pyANI [37,38], and OrthoANI was used for comparing the genomic similarity between the coding regions of the genomes [39]. For the pangenomic analysis, we downloaded *S. schleiferi* (n = 50) and *S. coagulans* (n = 182) assemblies from NCBI (https://www.ncbi.nlm.nih.gov/, accessed on 20 December 2023). The assemblies were subjected to Prokka annotation [40] and the Roary pangenome pipeline [41]. 

Phylogenetic relationships between *S. schleiferi* isolates from this study and previously sequenced, assembled, and annotated *S. schleiferi* isolates were determined using CSI Phylogeny 1.4 [42] (Call SNPs and Infer Phylogeny) using the *S. schleiferi* type strain MGYG-HGUT-01437 (GCA_902374935.1) as the reference genome using the default settings [minimum depth at single-nucleotide polymorphism (SNP) positions: 10×; minimum relative depth at SNP positions: 10 bp; minimum SNP quality: 10%, minimum distance between SNPs (prune): 30; minimum read mapping quality: 25 and minimum Z-score: 1.96].

## 3. Results

### 3.1. Genomic Features of Human Staphylococcus schleiferi and Staphylococcus coagulans

The genome size, GC content, predicted coding sequences, and predicted RNAs of the three *S. schleiferi* isolates (*S. schleiferi* 192, *S. schleiferi* 205, and *S. schleiferi* 214), two *S. coagulans* (*S. coagulans* 191 and *S. coagulans* 196) submitted to diagnostic laboratories from human cases in the USA, and the *S. schleiferi* type strain ATCC43808 isolated from a human patient in France are listed in (Table 2). 

### 3.2. Type-Based Species Clustering

**Figure 1:**
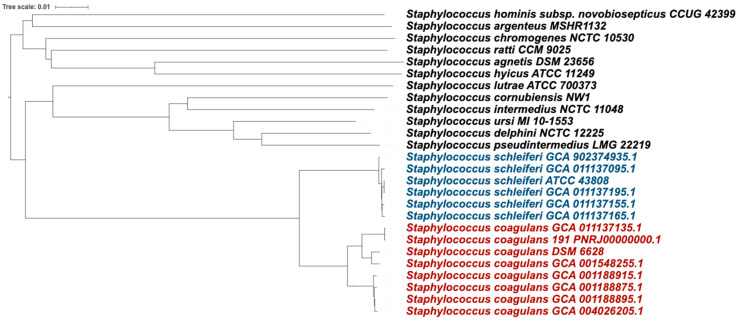
Tree inferred with FastME 2.1.6.1 [10] from GBDP distances calculated from genome sequences. The branch lengths are scaled in terms of GBDP distance. The tree was rooted at the midpoint [33].

The Type (Strain) Genome Server was used for genome-based analysis because of the high quality of its comprehensive, curated database of type strains [28]. This analysis yielded 18 species clusters, and *S. coagulans* 191 and *S. coagulans* 196 were classified as *S. coagulans,* while *S. schleiferi* 192, *S. schleiferi* 205, *S. schleiferi* 214, and *S. schleiferi* type strain ATCC43808 were classified as *S. schleiferi* (Figure 1). 

**Figure 2:**
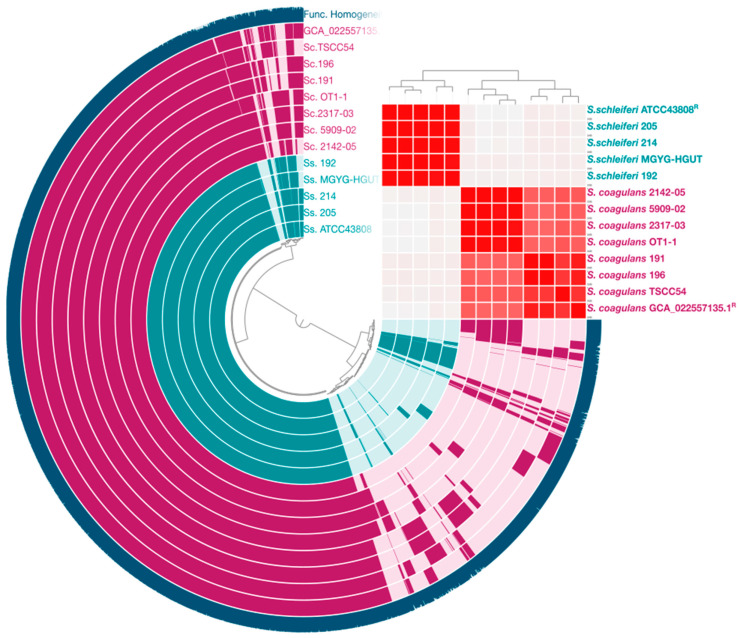
Static images from the Anvi’o-8 interactive display for the *S. schleiferi* and *S. coagulans* dataset.

Pangenome analysis was generated with Anvi’o-8. The layers represent individual genomes organized by their phylogenomic relationships based on single-copy core genes (Figure 2). In the layers, dark colors indicate the presence of a gene cluster and light colors its absence. The average nucleotide identity (ANI) heatmap illustrates the genetic relatedness of *S. schleiferi* and *S. coagulans*, with nucleotide identities ranging between 95% and 100% (Figure 2). Specifically, *S. coagulans* 191 and *S. coagulans* 196 form a cluster with other closely related strains, including *S. coagulans* 2317-03, *S. coagulans* OT1-1, *S. coagulans* 5909-02, *S. coagulans* TSCC54, *S. coagulans* strain 1031336 reference genome GCA_022557135.1, and *S. coagulans* 2142-05 (Figure 2). On the other hand, *S. schleiferi* 192, *S. schleiferi* 205, *S. schleiferi* 214, and the *S. schleiferi* type strain ATCC43808 are grouped together and share a high genetic similarity with *S. schleiferi* MGYG-HGUT-01437 (Figure 2). 

The clustering dendrogram shows the relationship of contigs based on their sequences and distribution across samples. ANI heatmap results for *S. schleiferi* and *S. coagulans* vary between 95 and 100 nucleotide identities %. The phylogenomic tree was reconstructed using single-copy genes. In the layers, dark colors indicate the presence of a gene cluster and light colors its absence. R stands for reference genomes that were used in the analysis.

**Figure 3:**
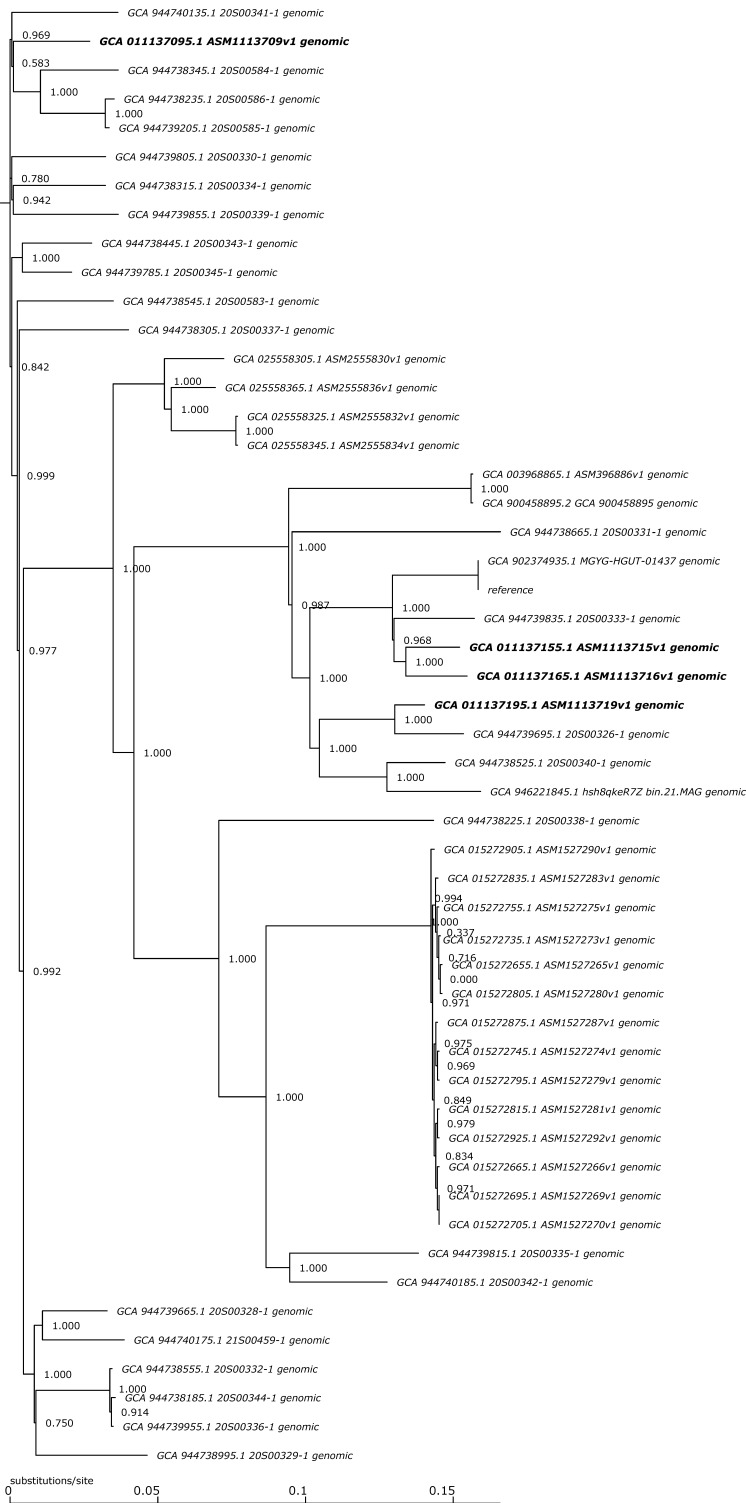
Phylogenetic tree based on the SNP analysis conducted in CSI Phylogeny 1.4 comparing *S. schleiferi* 192, *S. schleiferi* 205, *S. schleiferi* 214, and *S. schleiferi* ATCC 43808^T^ genomes with 46 publicly available *S. schleiferi* genomes.

*S. schleiferi* 192, *S. schleiferi* 205, *S. schleiferi* 214, and *S. schleiferi* ATCC 43808^T^ were compared with the 46 publicly available *S. schleiferi* genomes using an SNP-based phylogenetic analysis (Figure 3). The percentage of the reference genome contained within all isolates was found to be 93.19%, and 2,310,197 single nucleotide polymorphisms (SNPs) positions were found in the analyzed genomes (Figure 3).

**Figure 4:**
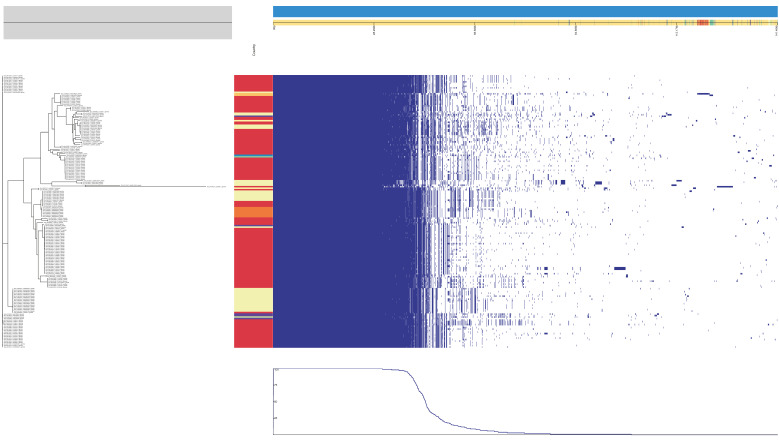
Pangenome analysis of 182 *S. coagulans* genomes using Roary. The left panel represents the Bayesian phylogenetic tree based on *S. coagulans* core gene single-nucleotide polymorphisms (SNPs) and Roary matrix. The right panel represents the matrix where the accessory and core genes were either present or absent. Isolates from different countries are color-coded as follows: Netherlands: #e4524a, USA: #5e4fa2, Scotland: #f2f9a9, Thailand: #f67d47, South Korea: #bce6a0, United Kingdom: #75c8a4, Japan: #1e90bf, Russia: #fdbf6c, and India: #fdeea1.

**Figure 5:**
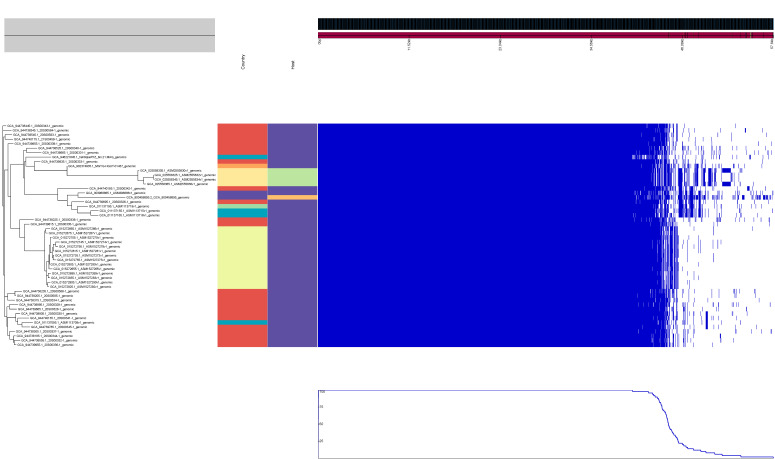
Pangenome analysis for 50 *S. schleiferi* genomes using Roary. The left side represents the Bayesian phylogenetic tree based on *S. schleiferi* core gene single-nucleotide polymorphisms (SNPs) and Roary matrix. The right side represents the matrix where the accessory and core genes were either present or absent. Isolates from different countries are color-coded as follows: Netherlands:#e4524a, Australia: #eef8a2, USA: #00a4bd, France: #a1d9a4, United Kingdom: #5e4fa2, Switzerland: #fde999 and Not provided: #fca55b. Isolates from different hosts are color-coded as follows: Homo sapiens: #5e4fa2, Camel: #bce6a0, and Not provided: #fdbf6c.

A genome-wide association study (GWAS) conducted using Roary and Scoary (Figure 4 and Figure 5) delineated species-specific genetic markers associated with *S. schleiferi* and *S. coagulans*. The comprehensive analysis revealed a distinct repertoire of genes unique to each species, with particular emphasis on the sialidase B (*nanB*) gene, Zn-dependent alcohol/formaldehyde dehydrogenase (*FrmA*), metal-dependent amidase/aminoacylase/carboxypeptidase (*AbgB*), membrane protease (*YdiL*), DNA glycosylase (*YcaQ*), and hypothetical protein 24 exclusively linked to *S. schleiferi* isolates and the chromate transport protein (*chrA*) gene identified as a unique feature of *S. coagulans* isolates.

### 3.3. Nucleotide Sequence Accession Numbers

The whole genome sequences of humans *S. schleiferi*, *S. coagulans* 191, *S. schleiferi* 192, *S. coagulans* 196, *S. schleiferi* 205, *S. schleiferi* 214, and *S. schleiferi* ATCC 43808^T^, have been deposited at DDBJ/ENA/GenBank under the accession numbers **PNRJ00000000**, **POVG00000000**, **POVH00000000**, **POVI00000000**, **POVJ00000000** and **POVK00000000**, respectively.

The *S. coagulans* 191, *S. schleiferi* 192, *S. coagulans* 196, *S. schleiferi* 205, *Staphylococcus schleiferi* 214, and *S. schleiferi* ATCC 43808^T^ accessions described in this paper are **PNRJ01000000**, **POVG01000000**, **POVH01000000**, **POVI01000000**, **POVJ01000000** and **POVK01000000**, respectively.

## 4. Discussion

In this study, *S. schleiferi* and *S. coagulans* were found to be distinct from each other while also phylogenetically separated from other species on their own branch. This finding, along with difficulties distinguishing them from each other and from other pathogens, in clinical laboratories, justifies our in-depth analysis and comparison of their genomic sequences. 

Most staphylococcal studies have focused on the pathogenesis, the development of vaccines, and epidemiological typing tools for *S. aureus* and *S. pseudintermedius.* Whereas other less studied staphylococcus species, such as *S. schleiferi* and *S. coagulans*, cause fewer infections, they are nonetheless important pathogens. *S. coagulans* may be underrecognized and underdiagnosed because, as Gram positive cocci that are coagulase positive, they may be misidentified as *S. aureus* or other staphylococci. Furthermore, *S. schleiferi* has a high incidence of methicillin resistance, with some reports indicating it may be as high as 57% in canine clinical isolates. Although *S. schleiferi* and *S. coagulans* are primarily associated with disease in dogs and occasional zoonotic infections, they have a global distribution and are also present on mink, birds, racoons, foxes, horses, goats, seals, and squirrels. 

The goal of this research was to improve our understanding of the *S. schleiferi* and *S. coagulans* genomic landscapes, lay the groundwork for the development of an effective *S. schleiferi* and *S. coagulans* vaccine, and advance our overall capabilities to develop staphylococcal vaccines in naturally infected hosts. The comparative genomic study identified species-specific indicators, including the chromate transport protein (*chrA*) gene, which is only present in *S. coagulans* isolates. This finding is consistent with previous studies and provides a new foundation for discriminating between the two species. Genes found in *S. schleiferi* but not *S. coagulans* isolates include the sialidase B (neuraminidase B) (*nanB*) gene, Zn-dependent alcohol/formaldehyde dehydrogenase (*FrmA*), metal-dependent amidase/aminoacylase/carboxypeptidase (*AbgB*), membrane protease (*YdiL*), DNA glycosylase (*YcaQ*), and the hypothetical protein 24 gene. In addition to their use in the development of molecular tests, such as PCR, to discriminate between the species, these putative findings may represent some important virulence factors that can be targeted with vaccines or methods of gene regulation to neutralize their activity. Neuraminidase B plays an important role in the evasion of mucus binding in *Streptococcus pneumoniae* [43]. It controls expression of the neuraminidase A gene (*nanA*) through the release of sialic acid that upregulates *nanA* expression. Hammond et al. postulated that NanA and NanB act together to allow *S. pneumoniae* to evade mucosal defenses. 

Formaldehyde is produced by *S. aureus* via the alternative heme degradation pathway to acquire iron [44]. *FrmA* is involved in formaldehyde detoxification; however, its role in *S. schleiferi* infections and iron acquisition has not been studied, although iron uptake is associated with virulence in Staphylococcus [45].

*AbgB* encodes a carboxypeptidase that is a member of the M20 family of metallopeptidases [46]. This family includes a methicillin-resistant *S. aureus* antibiotic resistance factor, *p*-aminobenzoyl-glutamate hydrolase. 

*YdiL* encodes a membrane protease. Proteases serve multiple functions in protecting Staphylococcus from their hosts’ immune defenses. They inhibit phagocyte-mediated killing, inactivate components of the immune system, and interfere with epithelial defenses [47].

Mitomycins, such as mitomycin C, are bifunctional alkylators that are able to kill bacteria by crosslinking complementary strands of DNA and preventing DNA replication. They accomplish this indirectly through the reduction in quinone. *YcaQ* protects bacteria from crosslinking compounds by unhooking the crosslinked DNA [48]. 

The ChrA protein, whose gene was identified in *S. coagulans*, serves as an efflux pump. It transports chromate from bacteria but also has a number of homologs, many of which are not well characterized [49]. Given the importance of efflux pumps in antimicrobial resistance, especially in biofilms, it is possible that ChrA or a homolog play this role in *S. schleiferi* [50].

These genes and their associated products represent potentially important virulence factors and mechanisms of resistance. Additional research will be required to determine what role, if any, these proteins play in staphylococcal infections and effective vaccines. Additionally, our study has resulted in the identification of numerous hypothetical proteins, highlighting the complexity of the genetic landscape in both *S. schleiferi* and *S. coagulans*. These uncharacterized proteins constitute a viable area for future research. Further characterization and investigation of these putative proteins (Table S1) has the potential to reveal novel processes, virulence factors, or adaptive properties that may contribute to the distinct characteristics of these Staphylococcus species. This research not only improves our understanding of the genetic differences between *S. schleiferi* and *S. coagulans*, but it also underscores the importance of continuing to explore the undiscovered parts of their genomes.

The identification of these unexplored proteins may provide a better understanding of the biological complexities that define the pathogenic potential and adaptive strategies of *S. schleiferi* and *S. coagulans*. Despite the useful insights garnered from this study, it is important to recognize its limitations. One significant limitation is the small number of *S. schleiferi* isolates included in the study. The majority of these isolates came from similar geographical areas, primarily the Netherlands and Australia. This geographical bias may have an impact on the overall diversity captured in the study, thereby limiting the generalizability of the findings to a broader global setting. Furthermore, the majority of the *S. coagulans* isolates were obtained from Scotland and the Netherlands. This geographic concentration poses challenges regarding the representation of the global genetic diversity within *S. coagulans* populations. Bacterial populations can vary significantly depending on factors such as host populations, the environment, and local selective pressures. As a result, the observed species-specific genes may not represent the whole range of genetic adaptations that may exist in *S. schleiferi* and *S. coagulans* populations from different geographical areas. The identified species-specific genes should be functionally characterized in the future, with an emphasis on their roles in pathogenesis and host interactions.

Comparative transcriptomics and proteomics could provide insights into gene expression patterns and protein functions, further elucidating the adaptive strategies employed by these Staphylococcal species. 

## 5. Conclusions

This study adds to the field of bacterial genomics by identifying species-specific genes in *Staphylococcus coagulans* and *Staphylococcus Schleifer*. The identified genes act as molecular signatures, potentially providing the basis for improved diagnostic techniques. The study also emphasizes the need to consider the pangenome rather than a single reference genome in order to gain a more complete knowledge of genetic variation within and between species. While this work provides useful insights into the genomes of *S. schleiferi* and *S. coagulans*, the limitations due to geographic bias highlight the necessity of ongoing research efforts that include a more diverse and internationally representative collection of isolates.

## Figures and Tables

**Table 1:** Accession numbers for the genome sequences retrieved from GenBank, utilized in the Anvio-8 analysis of *S. schleiferi* and *S. coagulans isolates*.

Isolate	GenBank Accession Number
*S. schleferi* MGYG-HGUT-01437	GCA_902374935.1
*S. coagulans* 2317-03	GCA_001188915.1
*S. coagulans* OT1-1	GCA_004026205.1
*S. coagulans* 5909-02	GCA_001188875.1
*S. coagulans* TSCC54	GCA_001548255.1
*S. coagulans* reference genome	GCA_022557135.1
*S. coagulans* 2142-05	GCA_001188895.1

All genomes were downloaded from NCBI (ftp://ftp.ncbi.nlm.nih.gov, accessed on 20 December 2023).

**Table 2:** Genomic features of human *Staphylococcus schleiferi*.

Strain	WGS Accession No *	No of Contigs	N50 (bp)	Genome Length (bp)	G+C Content (%)	Predicted Coding Sequences	Predicted RNAs
*Staphylococcus coagulans* 191	PNRJ00000000	51	138,893	2,508,133	35.73	2294	72
*Staphylococcus schleiferi* 192	POVG00000000	102	59,786	2,452,487	35.87	2203	74
*Staphylococcus coagulans* 196	POVH00000000	56	110,279	2,508,604	35.74	2299	74
*Staphylococcus schleiferi* 205	POVI00000000	92	56,958	2,468,342	35.92	2218	76
*Staphylococcus schleiferi* 214	POVJ00000000	94	57,247	2,469,699	35.92	2216	76
*Staphylococcus schleiferi* ATCC 43808^T^	POVK00000000	88	56,938	2,469,638	35.92	2218	73

* WGS—whole genome sequence.

## Data Availability

The data presented in this study are openly available in GenBank as noted.

## Supplementary Materials

The following supporting information can be downloaded at: https://www.mdpi.com/article/10.3390/genes15030284/s1, Table S1: Gene clusters identified in *S. schleiferi* and *S. coagulans* isolates.
